# Screening Reliable Reference Genes for RT-qPCR Analysis of Gene Expression in *Moringa oleifera*

**DOI:** 10.1371/journal.pone.0159458

**Published:** 2016-08-19

**Authors:** Li-Ting Deng, Yu-Ling Wu, Jun-Cheng Li, Kun-Xi OuYang, Mei-Mei Ding, Jun-Jie Zhang, Shu-Qi Li, Meng-Fei Lin, Han-Bin Chen, Xin-Sheng Hu, Xiao-Yang Chen

**Affiliations:** 1 State Key Laboratory for Conservation and Utilization of Subtropical Agro-Bioresources, South China Agricultural University, Guangdong, 510642, China; 2 Guangdong Key Laboratory for Innovative Development and Utilization of Forest Plant Germplasm, South China Agricultural University, Guangdong, 510642, China; 3 Guangdong Province Research Center of Woody Forage Engineering Technology, South China Agricultural University, Guangdong, 510642, China; 4 College of Forestry and Landscape Architecture, South China Agricultural University, Guangzhou, Guangdong, 510642, China; Hainan University, CHINA

## Abstract

*Moringa oleifera* is a promising plant species for oil and forage, but its genetic improvement is limited. Our current breeding program in this species focuses on exploiting the functional genes associated with important agronomical traits. Here, we screened reliable reference genes for accurately quantifying the expression of target genes using the technique of real-time quantitative polymerase chain reaction (RT-qPCR) in *M*. *oleifera*. Eighteen candidate reference genes were selected from a transcriptome database, and their expression stabilities were examined in 90 samples collected from the pods in different developmental stages, various tissues, and the roots and leaves under different conditions (low or high temperature, sodium chloride (NaCl)- or polyethyleneglycol (PEG)- simulated water stress). Analyses with geNorm, NormFinder and BestKeeper algorithms revealed that the reliable reference genes differed across sample designs and that ribosomal protein L1 (*RPL1*) and acyl carrier protein 2 (*ACP2*) were the most suitable reference genes in all tested samples. The experiment results demonstrated the significance of using the properly validated reference genes and suggested the use of more than one reference gene to achieve reliable expression profiles. In addition, we applied three isotypes of the superoxide dismutase (*SOD*) gene that are associated with plant adaptation to abiotic stress to confirm the efficacy of the validated reference genes under NaCl and PEG water stresses. Our results provide a valuable reference for future studies on identifying important functional genes from their transcriptional expressions via RT-qPCR technique in *M*. *oleifera*.

## Introduction

*Moringa oleifera* Lam., belonging to a single-genus family Moringaceae, is a fast-growing tree species and widely distributed in the tropical and subtropical regions [1]. This species, with a great economic value for food and medical industry [1, 2, 3, 4], has gained interest globally, especially in the developing countries because of its rich nutrition in various organs (in particular, the mostly used leaves and seeds). However, the potential utility of *M*. *oleifera* is not fully explored owing to the restriction of effective technologies or the lack of high-yielding varieties in seed production or in total biomass. Currently, besides the physiological and medical studies, researchers start to investigate genetic diversity of *M*. *oleifera* using molecular markers, and to develop marker-assistant selection (MAS) for genetic improvement [5]. A reference genome of *M*. *oleifera* is now publically accessible [6]. The transcriptomes from *M*. *oleifera* leaves were sequenced in our lab, which provided a well-assembled and annotated sequence database for gene function research.

One objective in our breeding program is to search for the genes associated with important agronomical traits. At the transcriptional level, the RT-qPCR technique provides an effective approach for assessing gene expression and for rapidly quantifying mRNA transcripts [7, 8, 9, 10]. Thus, this technique helps to identify the function of important genes in agronomy. However, the technique requires reliable reference genes in designing RT-qPCR experiments, which is crucial for accurately interpreting the expression of target genes [9]. Previous experiments showed that use of the unstably expressed reference genes could produce a biased experiment result and a false-positive conclusion [11, 12, 13, 14]. The significance of using reliable reference genes is also reflected from studies on the expression stability of various genes in experiments in different plant species, such as *Arabidopsis* [15], soybean [16], tomato [17], rice [18] and tobacco [19]. An ideal reference gene, in principle, should possess a property of a general cell function and a relatively invariable expression in differential tissues, or in different developmental stages, or under different experiment conditions [20, 21, 22]. Housekeeping genes or endogenous control genes were conventionally used for reference genes in RT-qPCR analysis, but some studies showed that these reference genes could exhibit great variations in expression under different experiment conditions [13, 23]. For instance, the glyceraldehyde-3-phosphate dehydrogenase (*GAPDH*) gene exhibited unstably expressions in papaya during its storage at different temperatures [24], and *actin* was inappropriate as a reference gene under the salinity stress in potato [25]. Because universal reference genes are not available in plants [26], it is necessary to search for the reliable reference genes that are suitable for experiment study in *M*. *oleifera*.

Earlier studies with *M*. *oleifera* presumed *GAPDH* and alpha tubulin (*TUA*) as reference genes in RT-qPCR, but did not examine the expression stabilities of these two genes under different conditions [27]. Here, we selected 18 candidate genes from the transcriptome database of *M*. *oleifera* generated in our lab, and evaluated their expression stabilities. These candidate reference genes were *GAPDH*, phosphoenolpyruvate carboxylase (*PEPC*), acyl carrier protein 1 (*ACP1*) and 2 (*ACP2*), ubiquitin-conjugating enzyme (*UBCE*), alpha tubulin 1 (*TUA1*) and 2 (*TUA2)*, ribosomal protein L1 (*RPL1*) and L2 (*RPL2*), malate dehydrogenase 1 (*MDH1*) and 2 (*MDH2*), actin (*ACT*), ubiquitin extension protein (*UEP*), translation elongation factor 1 (*EF1*) and 2 (*EF2*), beta-tubulin (*TUB*) and cyclophilin 1 (*CYP1*) and 2 (*CYP2*). The purpose of this study was to identify more reliable reference genes for normalizing target gene expressions via RT-qPCR. Experimental samples were collected under different conditions, including the pods in different developmental stages, various tissues, the leaves under low and high temperature, and the roots under water stresses (NaCl and PEG-simulated). Furthermore, to evaluate the usefulness of the validated reference genes, we examined the expressions of three isotypes of the superoxide dismutase (*SOD*) gene under NaCl and PEG water stresses, including copper-zinc *SOD* (*Cu/Zn-SOD*), iron *SOD* (*Fe-SOD*), and manganese *SOD* (*Mn-SOD*). These genes were known to be involved in plant adaptation to environmental stresses [28, 29, 30].

In our data analysis, we applied three algorithms (geNorm [12], NormFinder [31] and BestKeeper [32]) to assessing reference genes and to identifying the most stably expressed genes under different conditions. In addition, these tools were also used to do normalization over multiple reference genes, which could enhance the robustness for expression quantifications. Our finally selected reference genes are suitable for identifying functional genes under different environmental conditions with *M*. *oleifera*.

## Materials and Methods

### Plant materials

Six experiments were designed for collecting samples (**Table 1**). Samples were obtained from an open plantation (E113°37ˊ N23° 16ˊ) and some indoor plants grown in a green house in South China Agricultural University (SCAU), Guangzhou, China. Pods in different developmental stages were obtained from the plantation. It generally took 15 to 20 days for pod elongation growth. Pod samples were collected on the 3rd, 8th, 13th, and 18th days after pod formation. Except the pods, all other samples were collected from the seedlings of 2.5 months old grown in a culture room (26°C, 60% relative humidity) with perlites in nursery pots. Experiment materials with consistent height and growth were selected, but samples free from serious defects were chosen randomly. Tissue samples were roots, stems, mature and young leaves. For the samples under low and high temperature, plants after cultivation in the culture room were placed in the 4°C and 40°C illumination incubator, respectively. Leaves were then separately harvested at the 0, 2, 6, 12, 24, and 48 hours after these two treatments. For samples under water stress, plants were treated with 100 mmolL^-1^NaCl and 50gL^-1^ PEG6000 solutions, respectively. Roots were separately collected in 0, 2, 6, 12, and 24 hours after these two treatments. All samples were frozen in liquid nitrogen immediately after collection and stored at -80°C for subsequent RNA isolations. There were 90 samples in total, including biological replicates (**Table 1**).

**Table 1:** Samples collected in different experiments.

**Designs**	**Tissues**	**Biological replicates**	**Sampling times**	**Sample size (replicates × times)**
Different developmental stages	Pods	3	4	12
Tissues	Roots, stem, mature leaves, young leaves	3	1	12
Chilling stress	Leaves	3	6	18
High temperature stress	Leaves	3	6	18
Water stress (NaCl)	Roots	3	5	15
Water stress (PEG)	Roots	3	5	15

### RNA isolation and cDNA synthesis

All RNA samples were extracted with plant total RNA kit (OMEGA^8^) according to the manufacturer’s protocol. Samples were treated with the DNase digestion attached in the RNA kit to avoid genomic DNA contamination. The quality and quantity of the RNA samples were measured with NanoDrop ND1000 (Thermo Scientific). RNA samples with the absorbance ratios at both 260/280 and 260/230 nm being around 2.0 were selected. The integrity was assessed through 1.5% agarose gel electrophoresis to select the DNA-free RNA samples with well-defined bands. We added 1.2ug RNA for 30uL reverse-transcription system, which was equivalent to 400ng RNA for 10uL reverse-transcription system. The PrimeScript II first Strand cDNA Synthesis Kit MIX (Takara) was used according to the manufacturer’s instructions. The synthesized cDNA was diluted 15 times before used as the template for RT-qPCR.

### Selection of candidate reference genes

Eighteen candidate reference genes from previous reports were selected to screen the most reliable reference genes for target gene expressions via RT-qPCR technique. All genes were selected from our transcriptome database obtained by the high throughput Illumina HiSeq™ 2000 sequencing platform (*Gene Denovo*, Guangzhou, China), including *GAPDH*, *PEPC*, *ACP1*, *ACP2*, *UBCE*, *TUA1*, *RPL1*, *MDH1*, *MDH2*, *ACT*, *UEP*, *EF1*, *TUA2*, *TUB*,*CYP1*,*CYP2*, *RPL2 a*nd *EF2*. Information of these genes is detailed in **Table 2**.

**Table 2:** Eighteen candidate reference genes, PCR amplification primers, melting temperature (Tm), amplification efficiencies (*E*), and correlation coefficients (*R*^2^) examined in *Moringa oleifera*.

**Genes**	**Abbreviation**	*E*	*R*^*2*^	**Primer sequences (Forward/reverse)**	Tm (°C)	**Amplicon length(bp)**
Glyceraldehyde-3-phosphate dehydrogenase	*GAPDH*	1.019	0.999	AGCACAACAAGAAGGCGATAC/ CGATGGCAATGGACGGAATAT	84.34	190
Phosphoenolpyruvate carboxylase	*PEPC*	0.974	0.998	AAGAACAAAAGGCACAGACCAAC/ GATCCCTACTTAAAGCAAAGACTC	86.41	297
Acyl carrier protein	*ACP1*	0.952	1.000	AAACTTCTCCCACTGATGCG/ TCTTCGTGTTCTCCGTCCC	83.54	219
Acyl carrier protein	*ACP2*	1.002	1.000	GAAACCAATGAGCACCCAGC/ GATGAATACCAGTCCACCGCAAC	80.80	87
Ubiquitin-conjugating enzyme	*UBCE*	1.028	0.994	TCTATTGTTGTATGATGGGTAATGTG/ GGAAGGCGAGAACTGGAA	82.38	123
Ubiquitin extension protein	*UEP*	1.042	0.999	AAAAACCGCATAAACAAAAAGAG/ GCCCTCCTCCAGTTCTACAAG	87.57	204
Malate dehydrogenase	*MDH1*	0.913	0.999	GATAATACACTGCTGATCTCGG/ TAAACTTTGAGGGCATCGTC	82.75	131
Malate dehydrogenase	*MDH2*	0.903	0.994	TAGAAACGCACTAATAAAGACAAAGG/ AGAGTGGACAATAGTTCAAGGGC	83.27	146
Actin	*ACT*	0.939	0.994	TGGAAAGTGTCAAAGTGGGG/ CGATAATAACAACAGTAATGGCAGC	80.92	101
Elongation factor	*EF1*	0.951	1.000	ATCTGGCTTCTCAACTTCTGTC/ CCTCTTCTCCCTAAAACCCTAG	83.61	157
Elongation factor	*EF2*	0.950	0.995	CGAAGATGAAGAGGTGGGAG/ GCACTTGCCAAGCCTTTC	84.51	237
Alpha tubulin	*TUA1*	0.977	1.000	CCCACATACACCAACCTCAAC/ ACATCAAGCAGCAAGCCAT	84.76	292
Alpha tubulin	*TUA2*	0.963	0.996	AGACTCAGCACCCACCTCCTC/ TGTTCTCCCGCATTGACCAC	85.69	158
Beta-tubulin	*TUB*	0.994	0.996	AGTGTAATGCCCCTTAGCC/ CCAAGTTCTGGGAAGTAGTCTGT	85.86	269
Cyclophilin	*CYP1*	0.988	0.999	GAACTTGGAGCCGTAGATGG/ CCCGTTGGGCGTGTCGTTA	89.08	225
Cyclophilin	*CYP2*	0.958	0.996	TCTTTCTTGATTCACCACCCACTTG/ CATCTTCGCTGGATACTGTCG	83.39	177
Ribosomal protein L	*RPL1*	1.041	0.997	TGCTCGTGAAGCCGTAAAG/ CAAACCCTGAAGCCTCTGC	84.84	135
Ribosomal protein L	*RPL2*	0.972	0.998	TTTGGCTGGTTCCTGTTTAT/ ACGGTACAAGCAATGTATCCTG	81.40	125

### Primer design and RT-qPCR conditions

All primers for RT-qPCR analysis were designed using software primer 5.0 and Oligo 7 according to the sequences of candidate reference genes. Primer pairs were synthesized by Sangon company (Guangzhou, China). Primer pairs with a single product and a correct size were selected through 2.5% agarose gel electrophoresis. Each amplicon was sequenced by Sangon company to ensure the correctness of specific sequence. RT-qPCR was conducted to further check the specificity of amplicon. The RT-qPCR reaction mixture system, which was operated in 96-well optical plates (Bio-Rad, Foster City, CA, USA), consisted of 2μL cDNA, 10μL SYBR Green PCR Master Mix(Takara), 0.3μL of 10uM forward primer, 0.3μL of 10uM reverse primer, and 7.4μL ddH_2_O to a final volume 20μL. RT-qPCR reaction was then conducted by the Roche LightCyler 480 system (Roche^10^) under the following condition: 30s at 95°C followed by 40 cycles (5s at 95°C, 30s at 60°C, 30s at 72°C).

Melting curves were generated and analyzed by following the procedure. PCR amplification efficiency (*E*) and correlation coefficient (*R*^2^) for each gene were calculated through the experiments of fivefold cDNA dilution series, with three technical replicates for each standard curve. Primer pairs with the *E-*values between 90% and 110% were considered. Apart from the calculation of *E* and *R*^2^ using the fivefold cDNA dilution series, the synthesized cDNA samples from the six experiment designs were diluted 15 times before used as templates for RT-qPCR.

### Statistical analysis

Three commonly used statistical algorithms were applied to analyzing the expression stability of the 18 candidate reference genes under different experiment conditions: geNorm [12], NormFinder [31] and BestKeeper [32]. A threshold cycle (CT value), i.e. the number of cycles required for the fluorescent signal to exceed a specific detection threshold (removing noise signals) in the exponential phase of the PCR reaction, determined the expression levels of the tested candidate reference genes. A low CT value means a high level of gene expression. Each CT value in a single sample was from the average of three replicates.

Data inputs were different among the three algorithms. BestKeeper can directly deal with the raw CT values, without the need of data transformation. GeNorm and NormFinder need data transformation before proceeding calculations [12, 31]. All CT values were transformed into relative values. First, the "delta CT" value for each gene expression was obtained by subtracting the lowest CT value in a focal sample set. Hence, the lowest relative CT was 0 while others were greater than 0. Then, each relative CT value was transformed by 2^(-delta Ct)^. The gene with the highest 2^(-delta Ct)^ value (the minimum CT) was rescaled to 1, and was set as the reference gene. All others were rescaled with a reference to the reference gene, and hence were less than 1. Finally, the operated data file for each sample set was separately imported into geNorm and NormFinder.

The approach for determining the stably expressed genes is also different among the three algorithms. GeNorm ranked 18 candidate reference genes according to the estimated expression stability value (M) for each gene. The M value for each gene was calculated according to the average pairwise variation from all tested genes. The most stably expressed gene was the one with the lowest M value. GeNorm determined the optimal number of reference genes according to the relative value V_n_/V_n+1_, which measured the effect of adding one more reference gene on the normalization factor (the geometric mean of the expression values of the selected reference genes) [12]. An additional reference gene was included if it had a significant positive effect on the normalization factor. The value of V_n_/V_n+1_ below 0.15 was a cut-off value for determining the optimal number of reference genes (n).

NormFinder provided a stability value for each gene based on the variance analysis [31]. The gene with the lowest value had the most stable expression. BestKeeper provided the correlation (r) for a maximum of ten genes [32]. The gene whose r-value was closest to 1 was the most stably expressed gene.

### Normalization of *SOD genes*

Superoxide dismutase (*SOD*) catalyzes the dismutation reaction of superoxide radical (O_2_-) and protects plants from oxidative damage caused by reactive oxygen species (ROS), which is an adaptive response to environmental stresses [30]. In plants, there are three isotypes of the *SOD* gene based on their metal cofactors: *Cu/Zn-SOD*, *Fe-SOD*, and *Mn-SOD*. A study reported that the expression of the *SOD* gene significantly increased in pea under NaCl water stress [33]. The expression patterns of these genes under NaCl or PEG water stress were reported in previously published studies [34]. Here we selected these three isotype genes from our transcriptome database in *M*. *oleifera* to evaluate the validated candidate reference genes. Under the 100mM NaCl and 50gL^-1^ PEG water stresses, expressions of these three isotype genes were quantified using one or two most stably expressed reference genes. Note that a pre-experiment showed that a high PEG concentration (> 50gL^-1^) could make the seedlings of 2.5 month-old be severely wilted. The primer pairs (**S1 Table**) of the *SOD* gene were also verified before RT-qPCR analysis (**S1 Fig**).

## Results

### Amplification specificity and efficiency

**Fig 1:**
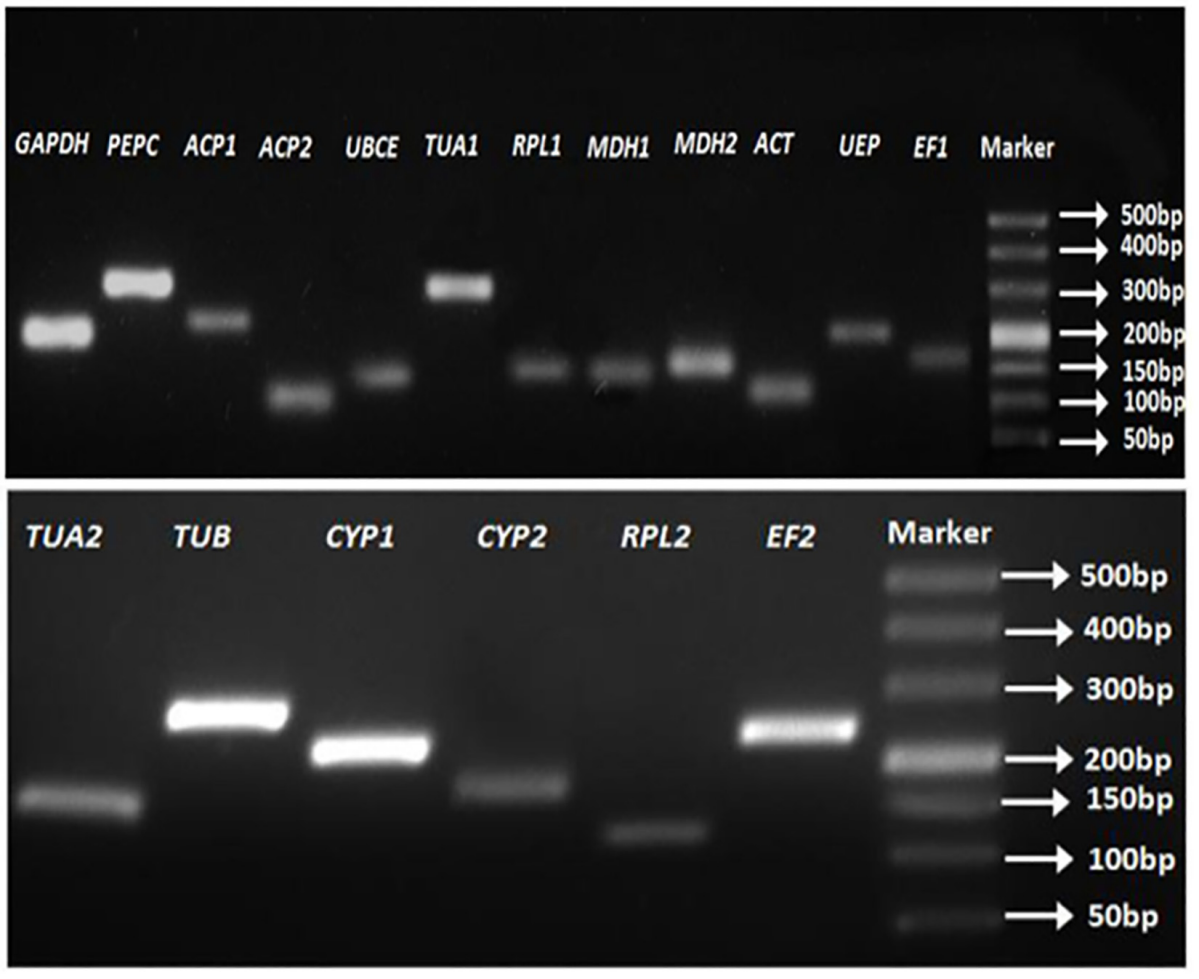
Amplifications of the eighteen candidate reference genes each with a different size estimated from markers.

Sequences of the eighteen candidate reference genes from *M*. *oleifera* transcriptome database were retrieved using BLASTX from NCBI and annotated according to the best blast result. Information about candidate reference genes and their primer pairs are summarized in **Table 2**. The results of sequencing and agarose gel electrophoresis (**Fig 1**) showed the specificity of 18 amplicons with correct sequences and fragment sizes. Verification of RT-qPCR revealed that each gene had a single peak in the melting curve (**S2 Fig**), indicating that the designed primers accurately amplified the target genes. PCR amplification efficiency and correlation coefficient of each primer pair were estimated through the simulated linear relationship between the log-transformed cDNA concentrations and the corresponding raw CT values from the fivefold cDNA dilution series experiments. PCR amplification efficiency ranged from 90.28% for *MDH2* to 104.18% for *UEP*, which was within the range from 90% to 110%. Correlation coefficients varied from 0.9941 for *ACT* and *UBCE* to 0.9999 for *TUA1* (**Table 2**).

### Expression profiles

**Fig 2:**
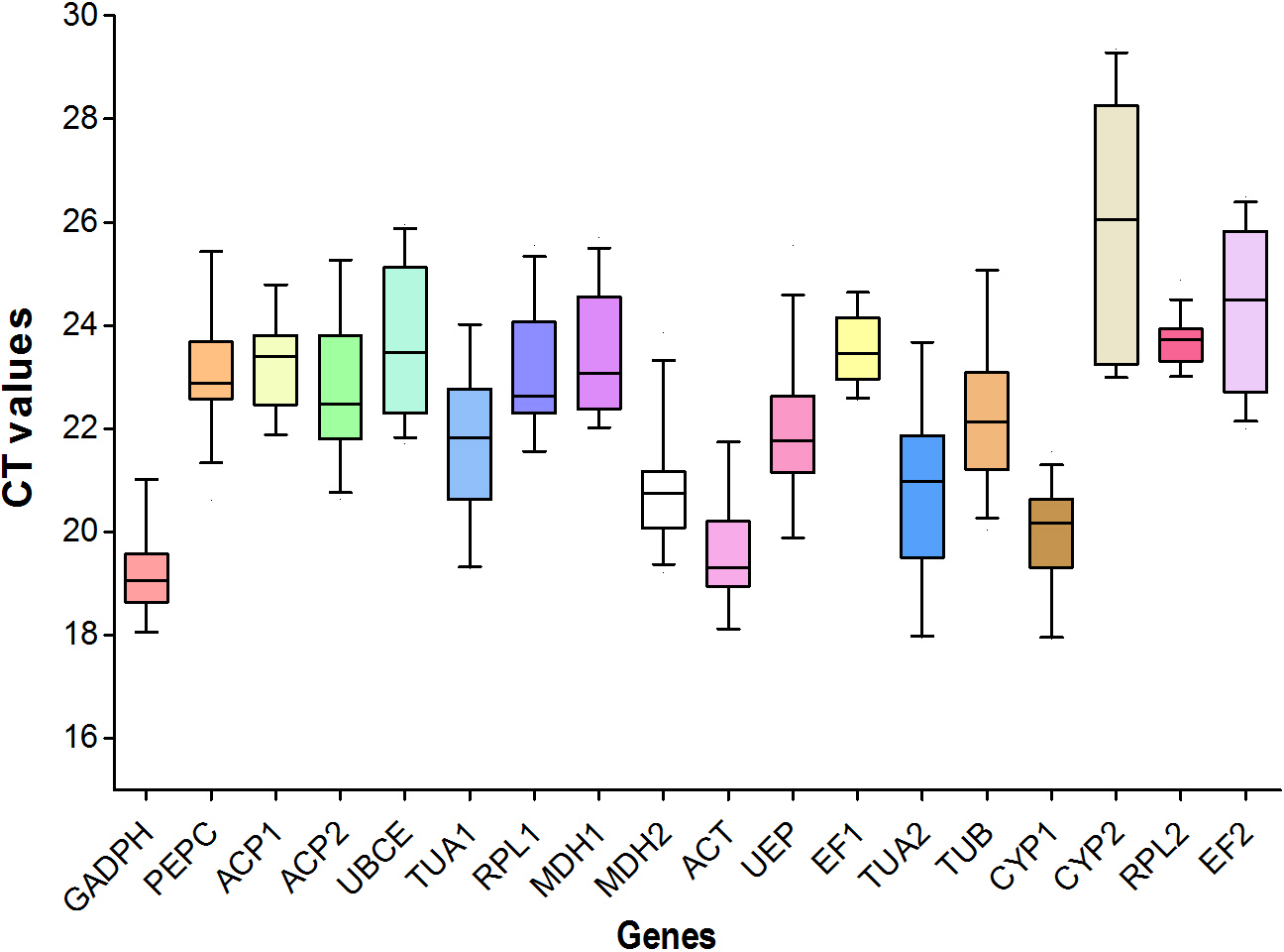
A boxplot for the CT values of eighteen candidate reference genes from RT-qPCR analysis. For each reference gene, the line inside the box is the median. The top and bottom lines of the box are the first and third quartiles, respectively. The top and bottom whiskers are the 5^th^ and 95^th^ percentiles, respectively.

Means and standard deviations of CT values are summarized for the 18 candidate reference genes (**Table 3; Fig 2**). A large difference existed among candidate genes in mean/median and variations. Genes *GAPDH*, *ACT* and *CYP1* exhibited higher levels of expressions than other genes in all samples, with the average CT values ranging from 19.18 to 19.97 cycles. *CYP2* (average CT = 25.97) exhibited the lowest level of expression. All other genes exhibited intermediate levels of expressions across all samples (**Table 3**).

**Table 3:** Means and standard deviations of the threshold cycle (CT value) for eighteen candidate reference genes in RT-qPCR analysis.

Genes	All samples	Tissues	Pods	4°C	40°C	NaCl	PEG	4°C and 40°C	NaCl and PEG
*GADPH*	19.18±0.78	19±0.39	18.86±0.53	19.39±0.22	20.27±0.84	18.73±0.41	18.49±0.39	19.83±0.74	18.61±0.40
*PEPC*	23.19±1.1	22.03±1.02	22.44±0.16	22.69±0.21	23.65±1	23.88±0.91	24.07±1.25	23.17±0.86	23.98±1.04
*ACP*1	23.22±0.86	22.49±0.47	22.01±0.18	23.82±0.22	24.27±0.5	23.12±0.42	22.92±0.59	24.04±0.44	23.02±0.49
*ACP*2	22.8±1.29	22.31±0.76	21.14±0.49	23.87±0.18	24.43±0.78	22.17±0.24	21.9±0.57	24.15±0.61	22.04±0.44
*UBCE*	23.69±1.39	24.12±0.85	22.71±0.3	25.13±0.66	24.96±0.76	22.46±0.49	22.13±0.47	25.05±0.69	22.30±0.49
*TUA*1	21.74±1.36	20.51±0.84	19.8±0.64	21.9±0.29	22.96±1.08	22.43±1.09	21.89±1.3	22.43±0.93	22.16±1.17
*RPL*1	23.15±1.14	22.69±0.87	21.77±0.24	24.07±0.15	24.65±0.69	22.55±0.07	22.34±0.31	24.36±0.56	22.44±0.24
*MDH*1	23.45±1.16	22.76±0.93	22.5±0.47	22.36±0.06	23.44±0.74	24.5±0.42	25.03±0.56	22.90±0.75	24.77±0.55
*MDH*2	20.82±1.03	20.11±0.45	19.62±0.35	21.05±0.17	22.37±0.97	20.52±0.32	20.49±0.48	21.71±0.96	20.50±0.38
*ACT*	19.63±0.95	19.18±0.58	18.33±0.26	20.18±0.15	20.95±0.72	19.32±0.15	19.08±0.15	20.56±0.64	19.20±0.19
*UEP*	21.97±1.25	21.44±0.7	20.27±0.46	22.65±0.15	23.66±1.04	21.64±0.27	21.25±0.58	23.16±0.88	21.44±0.47
*EF*1	23.52±0.67	23.39±0.36	22.86±0.27	24.16±0.16	24.31±0.32	22.95±0.34	23±0.29	24.23±0.26	22.97±0.30
*TUA*2	20.8±1.55	19.49±1.06	18.51±0.73	21.05±0.19	22.28±1.39	21.44±1.08	20.98±1.29	21.66±1.14	21.21±1.15
*TUB*	22.3±1.36	21.01±0.71	20.72±0.74	22.1±0.14	23.95±1.21	22.58±0.83	22.61±1.28	23.03±1.27	22.59±1.02
*CYP*1	19.97±0.92	18.95±0.28	18.74±0.92	20.29±0.26	20.56±0.9	20.45±0.43	20.16±0.75	20.43±0.64	20.31±0.60
*CYP*2	25.97±2.29	24.88±2.04	25.75±0.39	23.09±0.12	25.54±1.82	28.57±0.58	28.38±0.85	24.31±1.77	28.47±0.69
*RPL*2	23.69±0.43	23.63±0.4	23.45±0.36	23.84±0.21	24.06±0.44	23.56±0.46	23.43±0.43	23.95±0.35	23.49±0.43
*EF*2	24.31±1.49	23.75±1.76	24.41±0.27	22.69±0.1	23.57±1.24	25.88±0.12	25.97±0.45	23.13±0.96	25.93±0.32

Concerning the variation of gene expressions across all samples, *CYP2* and *TUA2* showed large CT variations, while *RPL2* and *EF1* showed small CT variations. Expressions for the rest of the candidate genes exhibited intermediate variations (**Table 3**; **Fig 2**).

The same gene exhibited expression variations in different sample designs (**Table 3**). There were no concordant patterns among all gene expressions in different experiment designs. Most genes (except *CYP*2, *EF*2, and *ACP*2) exhibited higher levels of expression in pods than in tissues or under the other conditions. Most genes except *UBCE* exhibited a higher level of expression under the low temperature (4°C) than under the high temperature (40°C). Gene *CYP*2 exhibited the lowest levels of expressions in tissues and pods samples or under the high temperature (40°C) and the water stress (NaCl and PEG). Gene *GADPH* had the lowest expression under the high temperature (40°C), but had relatively higher levels of expressions under water stress (**Table 3**).

The pooled CT values for water stress (NaCl and PEG) generally exhibited intermediate levels of expressions and variations, compared with the results under NaCl or PEG water stress alone. The same case occurred for the pooled CT values under temperature stress (4°C and 40°C) (**Table 3**).

Taken together, none gene was constantly expressed in all samples. It is necessary to further identify the appropriate reference genes for the data normalization under different experiment conditions.

### Expression stability

**Fig 3:**
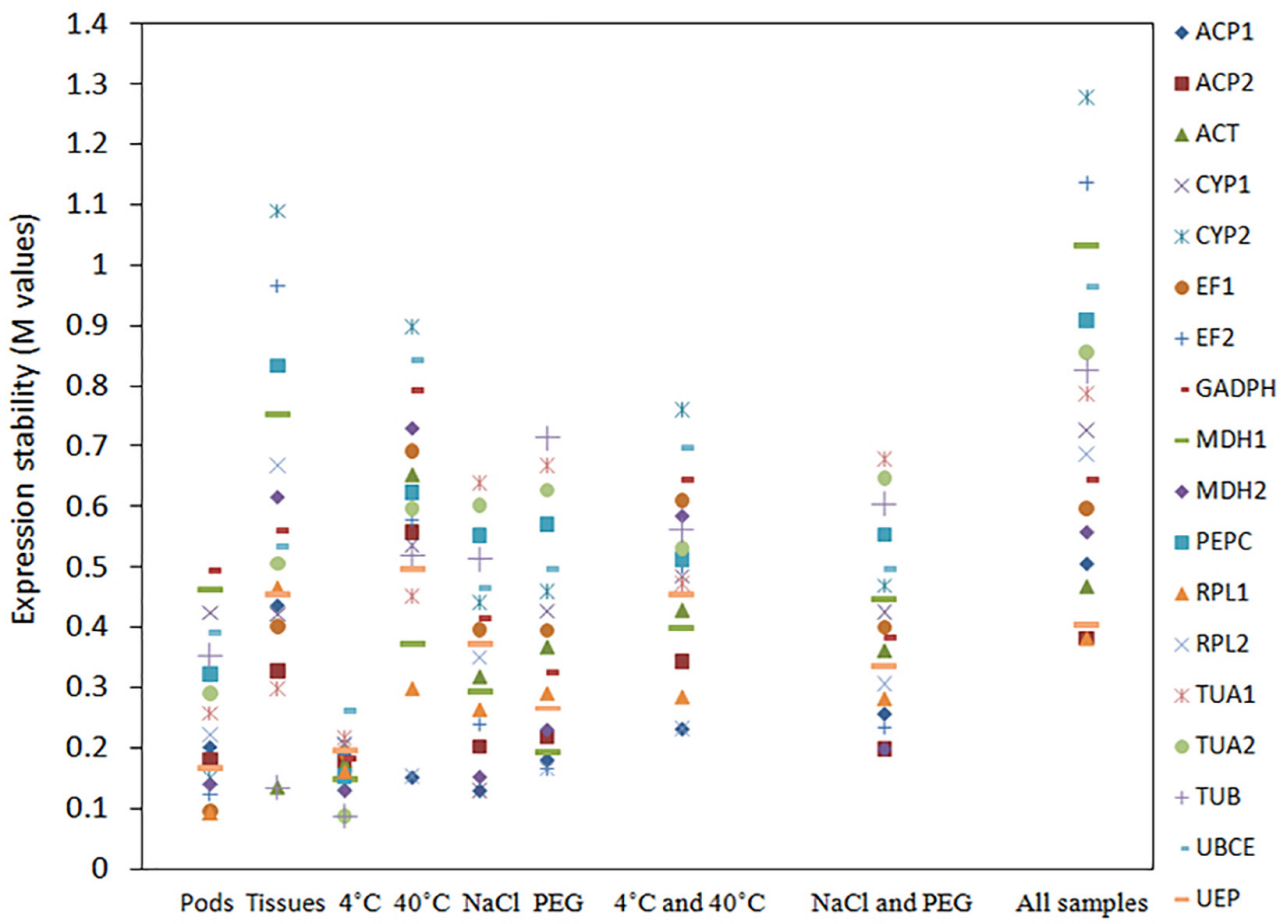
Expression stability for all candidate reference genes. The M values from the geNorm analysis were derived from a stepwise exclusion of the least stable reference genes. A lower M value indicated a more stable expression.

Analyses with geNorm, NormFinder and BestKeeper algorithms showed that the most stably expressed genes varied in different sample sets or with different algorithms. **Fig 3** shows the ordered gene expression stability from the analysis with geNorm under different conditions. The most stably expressed genes were *ACT* and *RPL1* (M = 0.092) for the pod samples in different developmental stages, *TUB* and *ACT* for various tissue samples (M = 0.135), *TUB* and *TUA2* for the roots under low temperature (M = 0.088), *RPL2* and *ACP1* for the roots under the high temperature (M = 0.152), *CYP1* and *ACP1* (M = 0.130) under NaCl water stress, and *EF2* and *RPL2* (M = 0.164) under PEG water stress. For pooled samples under both the low and high temperature, the most stably expressed genes were *RPL2* and *ACP1* (M = 0.232) (**Fig 3**). For pooled samples under both NaCl and PEG water stresses, the most stably expresses genes were *ACP2* and *MDH2* (M = 0.199) (**Fig 3**). For all pooled samples, *RPL1* and *ACP2* were the two most stably expressed genes (M = 0.382), and could be used as the reference genes for multiple samples (**Fig 3**). *CYP2* expression was the least stable among all gene expressions.

**Fig 4:**
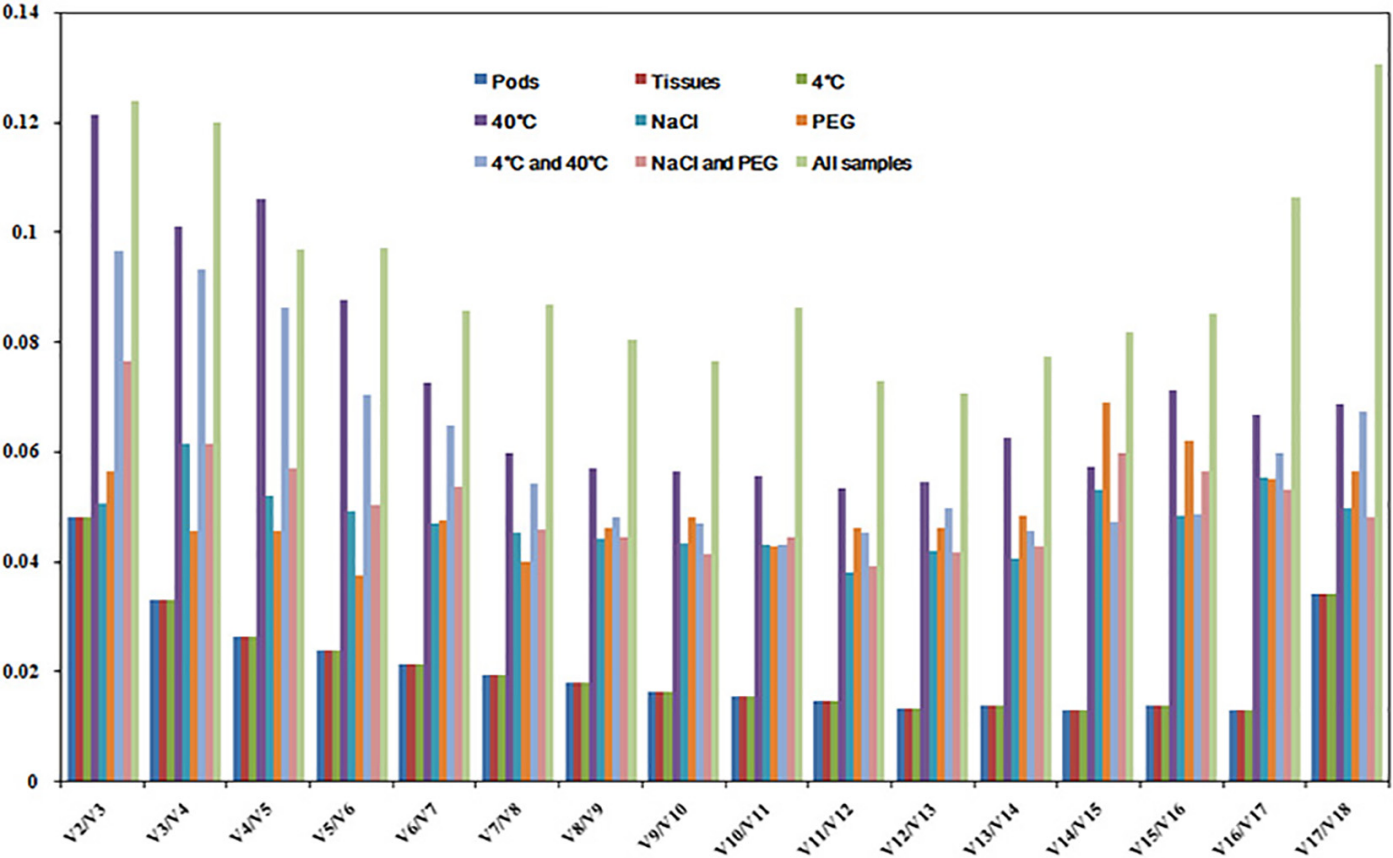
Pairwise variation (V) analysis among the candidate reference genes. The relative value V_n_/V_n+1_ was analyzed by geNorm to determine the optimal number of reference genes required for RT-qPCR data normalization. The relative value at V2/V3 indicated the optimal number of genes for normalization in each experiment design.

From the pattern of V_n_/V_n+1_ (**Fig 4**), we selected two reference genes (parsimony) in the nine sample sets for data normalization since their V_2_/V_3_ values were below 0.15, although an addition of more genes could further reduce the relative value *V*_n_/*V*_n+1_.

**Fig 5:**
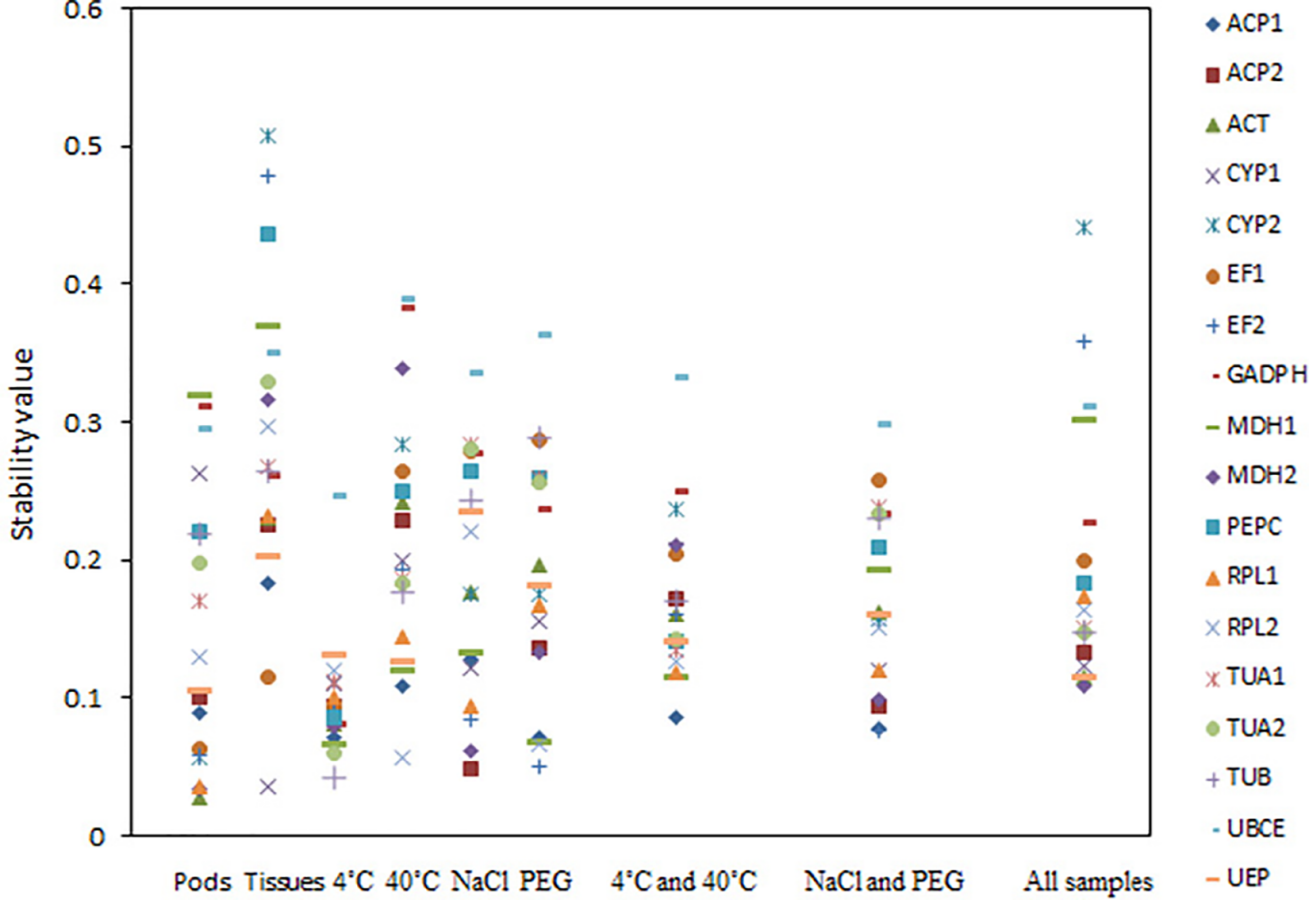
Expression stabilities for all candidate reference genes under different conditions. The stability values were derived from the analysis with NormFinder algorithm. A lower value indicated a more stable expression.

**Fig 5** shows the results from the analysis with NormFinder. The most two stably expressed genes were *ACT* (the stability value = 0.028) and *MDH*2 (0.034) for the pod samples in different developmental stages, *CYP*1(0.036) and *EF*1(0.115) for various tissue samples, *TUB* (0.043) and *TUA2* (0.06) for the roots under 4°C, *RPL2* (0.057) and *ACP1* (0.109) for the roots under 40°C, *CYP2* (0.049) and *MDH*2 (0.062) for the roots under NaCl water stress, and *EF2* (0.051) and *RPL2* (0.066) for the roots under PEG water stress. For pooled samples under the low and high temperature, the most stably expressed genes were *ACP*1 (0.086) and *MDH*1 (0.115). For pooled samples under both NaCl and PEG water stresses, the most stably expresses genes were *EF*2 (0.077) and *ACP*1 (0.078). For all pooled samples, *MDH*2 (0.109) and *UEP* (0.115) were the two most stably expressed genes.

Generally, the top six stably expressed genes were the same in most samples from both geNorm and NormFinder analyses. Two most stably expressed genes were the same from both NormFinder and geNorm analyses under the temperature (4°C and 40°C) and PEG water stresses. Two most stably expressed genes from the geNorm analysis were in the top six stably expressed genes from the NormFinder analysis, except *RPL1* that was ranked at the eleventh place in all sample sets and *TUB* that was ranked at the ninth place in various tissues from the NormFinder analysis. The most unstably expressed genes were generally the same in samples from the pods in different developmental stages or from various tissues. However, under the temperature (4°C and 40°C) and water stresses (NaCl and PEG), the most unstably expressed genes from the geNorm analysis were the third and fourth unstably expressed genes from the NormFinder analysis, respectively.

BestKeeper analyzed the top ten stably expressed genes that were derived from the geNorm analysis (**Table 4**). Unlike the results from the geNorm and NormFinder analyses, the ranking order from BestKeeper analysis exhibited larger variations, similar to the reports in cucumber and poplar species [14, 35]. For instance, the unstably expressed gene *EF2* under 40°C was ranked in the tenth and ninth places by geNorm and Normfinder analyses, respectively, but was ranked in the third place from the analysis with BestKeeper. For the less stably expressed genes, these three algorithms displayed a similar result.

**Table 4:** Ordering of the candidate reference genes according to their expression stability values calculated by BestKeeper

**Pods**		**Tissues**		4°C		40°C		**NaCl**		**PEG**		4°C and 40°C		**NaCl and PEG**		**All samples**	
**Genes**	**Values**	**Genes**	**Values**	**Genes**	**Values**	**Genes**	**Values**	**Genes**	**Values**	**Genes**	**Values**	**Genes**	**Values**	**Genes**	**Values**	**Genes**	**Values**
*ACT*	1.00	*ACP2*	0.97	*PEPC*	0.95	*TUB*	1.00	*MDH2*	0.90	*ACP2*	0.99	*TUA1*	0.98	*ACP2*	0.92	*UEP*	0.97
*UEP*	0.99	*TUA1*	0.97	*ACP1*	0.89	*TUA1*	0.98	*ACP2*	0.90	*EF2*	0.98	*UEP*	0.95	*RPL1*	0.87	*ACP2*	0.97
*CYP2*	0.98	*TUB*	0.94	*MDH2*	0.88	*EF2*	0.96	*ACP1*	0.84	*MDH2*	0.97	*EF2*	0.95	*UEP*	0.86	*RPL1*	0.96
*MDH2*	0.97	*TUA2*	0.93	*TUA2*	0.84	*MDH1*	0.96	*MDH1*	0.78	*MDH1*	0.94	*MDH1*	0.94	*MDH2*	0.79	*ACT*	0.95
*ACP2*	0.97	*RPL1*	0.93	*TUB*	0.69	*UEP*	0.95	*CYP1*	0.78	*ACP1*	0.90	*RPL1*	0.93	*EF2*	0.77	*ACP1*	0.94
*RPL1*	0.95	*ACT*	0.91	*ACP2*	0.56	*RPL2*	0.92	*RPL2*	0.73	*UEP*	0.89	*ACP1*	0.91	*RPL2*	0.74	*MDH2*	0.89
*EF1*	0.92	*EF1*	0.83	*RPL1*	0.50	*RPL1*	0.90	*EF2*	0.65	*RPL2*	0.88	*RPL2*	0.86	*ACP1*	0.70	*EF1*	0.84
*EF2*	0.91	*CYP1*	0.83	*ACT*	0.43	*ACP1*	0.90	*RPL1*	0.14	*RPL1*	0.83	*ACP2*	0.84	*GADPH*	0.60	*GADPH*	0.72
*RPL2*	0.72	*UEP*	0.83	*MDH1*	0.41	*CYP1*	0.87	*ACT*	-0.36	*GADPH*	0.60	*CYP1*	0.81	*ACT*	0.21	*CYP1*	0.69
*ACP1*	0.66	*ACP1*	0.80	*CYP2*	0.33	*ACP2*	0.83	*UEP*	-0.50	*ACT*	-0.13	*ACT*	0.79	*EF1*	0.13	*RPL2*	0.67

To further assess the gene expressions, the top-ten stably expressed genes from the geNorm analysis were scored from 1 to 10, representing the genes with the most stable to the most unstable expressions, respectively. Similarly, these ten genes from the analyses with both NormFinder and BestKeeper were also scored from 1 to 10 according to their previous ranking orders. The total score for each selected gene was calculated by summing these three scores from different algorithms. All the total scores were ordered. The gene with the lowest total score was thought to be the most stably expressed genes and marked as 1. The final results showed that the expression stability by the three algorithms was quite similar for the most stably and unstably expressed genes, even though their overall orders were different among them (**S2 Table**).

### Validation of reference genes

**Fig 6:**
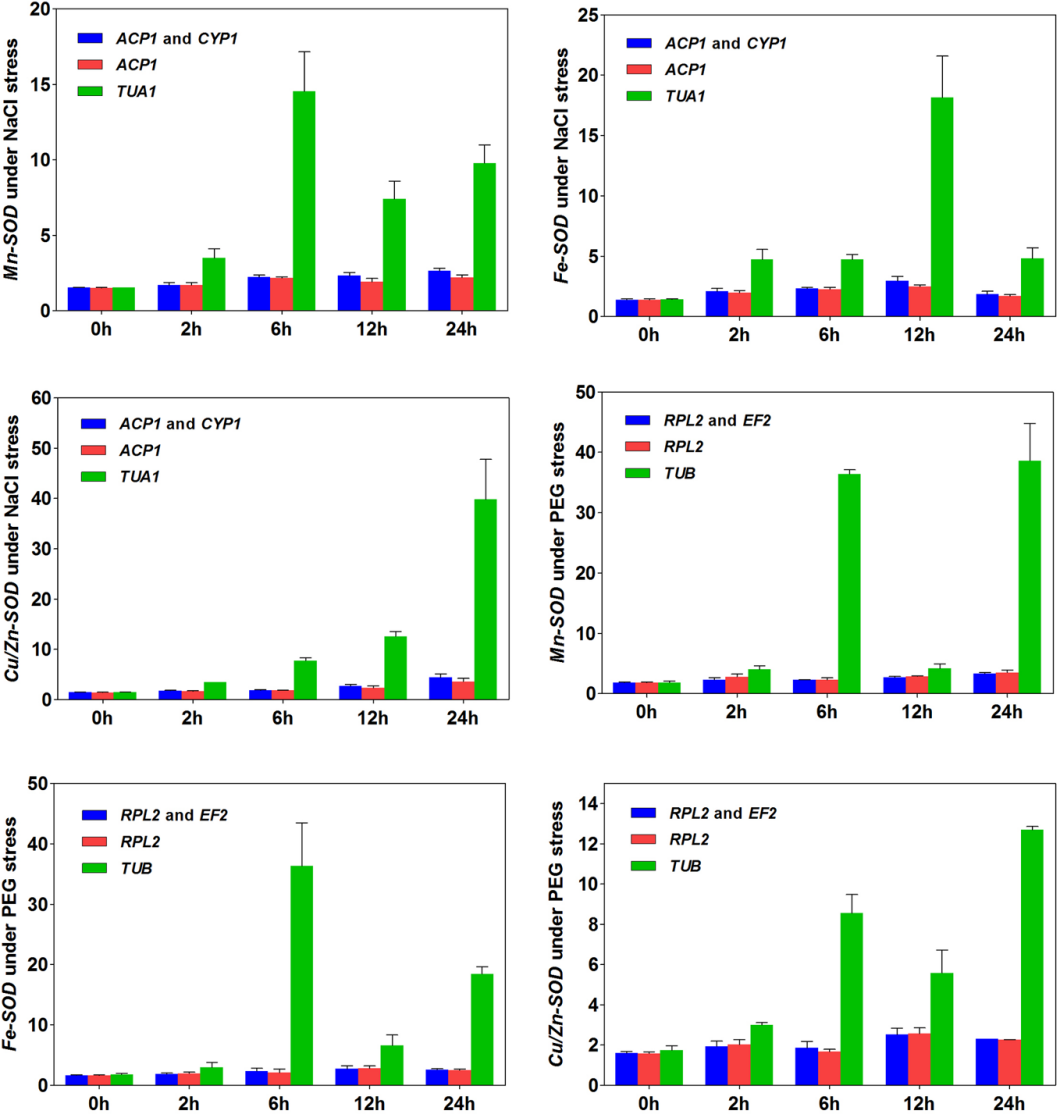
Expressions of three *SOD* genes using validated reference genes for normalization under NaCl and PEG water stresses.

Three isotypes of the *SOD* gene (*Cu/Zn-SOD*, *Fe-SOD*, and *Mn-SOD*) were employed to test the efficacy of the validated candidate reference genes under NaCl and PEG water stresses, respectively (**Fig 6**). The relative expressions of these three genes were quantified using one or two of the most stably and unstably expressed genes. Our results revealed that the expressions of three genes increased under NaCl water stress. Similar patterns among the three genes occurred when *ACP1* alone or the combination of *ACP1* and *CYP1* was used as the reference genes (**Fig 6**). Likewise, expressions of the three genes gradually increased under PEG water stress when *RPL2* alone or the combination of *RPL2* and *EF2* was used as the reference genes (**Fig 6**). In each case, the use of two reference genes under NaCl water stress (*ACP1* and *CYP1*) or under PEG water stress (*RPL2* and *EF2*) generally improved the quantification of the *SOD* gene expressions, compared with the results of using a single reference gene. However, the patterns changed when the least stably expressed gene *TUA1* under NaCl water stress or gene *TUB* under PEG water stress was used as the reference gene. This provided the evidence of biased quantifications when the unstably expressed genes were used as the reference genes in *M*. *oleifera*.

## Discussion

This study was to search for reliable candidate reference genes under different experiment conditions, with an aim at more accurately quantifying the expression of target genes in *M*. *oleifera* using RT-qPCR technique. The technique is effective for quantifying gene expression due to its sensitivity and accuracy, given that reliable reference genes are available for compensating the variations brought by sample differences [8]. A reliable reference gene should have a minimal variation in expression, whereas a gene of agronomical interest often changes its expression over the course of plant growth and development [21, 36, 37]. However, there are no universal reference genes suitable for different experiment conditions as many factors affect the stability of gene expressions [16, 26]. In addition, the number of selected reference genes can influence the quantification result for some apportioned experiments [12, 38]. There is evidence showing that the traditional strategy for normalization based on a single gene could generate biased results [39, 40]. These concerns have not been clarified with *M*. *oleifera*, and no any report is available on identifying and validating reference genes in this species.

We examined eighteen candidate reference genes from our transcriptome database. Samples were collected under different experiment conditions, including the pods in different developmental stages, various tissues, the leaves under low and high temperature, and the roots under NaCl and PEG water stresses. Three algorithms (geNorm [12], NormFinder [31] and BestKeeper [32]) were used to analyze gene expression stability. The results showed that the best candidate genes and the number of reference genes were: *ACT* and *RPL*1 for pods, *TUB* and *ACT* for tissues (stem, roots and leaves), *TUB* and *TUA2* for leaves under low temperature, *RPL2* and *ACP1* for leaves under high temperature, *CYP1* and *ACP1* for roots under NaCl water stress, *EF2* and *RPL2* for roots under PEG water stress, *RPL2* and *ACP1* for leaves under low or high temperature, *ACP2* and *MDH2* for roots under NaCl or PEG water stress, and *RPL1* and *ACP2* for all samples. Although different rankings were yielded among the three algorithms (**Figs 3** and **5**, **Table 4**), the most stably or unstably expressed candidate genes were essentially the same. We selected three isotypes of the *SOD* gene to evaluate the efficacy of validated reference genes, and provided the evidence that the number and the type of reference genes could affect the quantification of target gene expressions in *M*. *oleifera* (**Fig 6**). Such influences were verified to be remarkable when the stably or unstably expressed reference genes were used, indicating the importance of applying reliable reference genes to assessing functional genes in this species. Although the improvement of applying two reference genes over a single reference gene was not substantial for quantifying gene expressions, our results implied the necessity of applying multiple reference genes in some cases.

Conventional reference genes, such as *GAPDH*, *TUA*, *ACT*, *EF* and *CYP*, are commonly used for quantifying target gene expressions in many plant species. However, some studies reported that these reference genes were not always stably expressed when tested in different species or under various experiment conditions [8, 13, 41]. Genes *GAPDH* and *TUA* were used to quantify the expression of target genes in *M*. *oleifera* [27]. Our study revealed that *GAPDH* and *TUA1* were not the best choice in all tested experiments. *TUA2* only exhibited the best stability under low temperature. Our results suggested that more suitable reference genes other than *GAPDH* and *TUA* should be taken into account in future studies with *M*. *oleifera*. A previous study used *ACT* to normalize gene expression [13], but here we confirmed its feasibility in the pod samples from different developmental stages and in various tissue samples. However, this gene was not the best reference gene under low temperature, consistent with the results in a previous study with potato species under low temperature [25]. In addition, different levels of variations in the relative expressions of *EF* and *CYP* were observed in *M*. *oleifera* samples under different conditions, which also occurred in banana species [16]. Thus, similar to the conclusions from the studies in other species, the best candidate reference genes were not all in common under different conditions in *M*. *oleifera*.

The expression stability of a gene could be related to its biological function or its polymorphism in a species [42, 43, 44]. We commonly hold that housekeeping genes are often suitable for reference genes because they are more conservative in function or often have low genetic diversity (note that different alleles often exhibit different levels of expressions) [43, 45]. Here we confirmed that *ACT*, *TUB* and *RPL* had the best stability in more than one sample design. Genes *ACT* and *TUB* are the basal components for cytoskeleton that maintain the life activity for organisms, and exhibit conservative structure during the evolution, especially gene *ACT*. Gene *TUB* also presented a little variation in different tissues of *Jatropha curcas* and showed good stability under low temperature, indicating that *TUB* could be a good choice as a reference gene under some conditions [46]. *RPL* codes a ribosomal protein, the main component of ribosome and important for protein synthesis, is also highly conserved. *RPL1* and *RPL2* were suggested to be reference genes in our four sample designs. In addition, *ACP1* and *ACP2* were stably expressed in five sample designs, implying that *ACP* could be a reliable candidate reference gene in *M*. *oleifera*. A challenge for future studies is to examine which allele for each of these validated reference genes, provided that they are polymorphic, is dominantly and stably expressed under different conditions in *M*. *oleifera*.

In relation to the breeding program in *M*. *oleifera*, the reference genes obtained under various conditions could be used for accelerating selection for multiple objectives. To improve a breeding population of *M*. *oleifera* to adapt to different environmental conditions, such as different temperatures, soil moisture and salinization degree, it is an important step to identify and exploit suitable reference genes for quantifying the expression profiles of different target genes. As different tissues or the same tissue in different developmental stages contain different nutrient/medical components or active ingredients, the expression of relevant genes may be used to reveal the genetic basis of their synthetic mechanisms, which helps to better utilize *M*. *oleifera*. In addition, the genes related to biotic stresses, exogenous hormone treatments and disease resistance can also be explored. All these studies partly depend on the functional gene research besides the field experiment and genetic mating designs in breeding. RT-qPCR provides an effective technique to reveal the gene expression and relevant mechanisms at the transcriptional level. The type and the number of reference genes obtained in this study could be applied to designing breeding programs to explore multiple functional genes associated with different target traits [8, 12].

The algorithms used by geNorm [12], NormFinder [31] and BestKeeper [32] are currently used for analyzing candidate reference genes in RT-qPCR experiments, including selecting reference genes and assessing their expression stabilities. GeNorm, not NormFinder and BestKeeper, can be used to determine the number of reference genes [12]. As indicated in our study, the difference in ranking order existed among the outputs of these three algorithms [31]. Orders of the stably and unstably expressed reference genes were similar among most samples, especially the order based on the analysis with geNorm and on the total score (**S2 Table**). There was a relatively large difference between geNorm and BestKeeper analyses, but a small difference between geNorm and NormFinder analyses. Such differences were also reported in previous studies [47]. Thus, when the discordant patterns occur from three algorithms under some specific experiment conditions, a comprehensive comparison is needed in determining the number and the type of appropriate reference genes.

Finally, it is of interest to briefly discuss the necessity of using single versus multiple reference genes. Previous studies often emphasize the use of a single reference gene for calibrating expressions of target genes. However, the common situation is that a single reference gene may exhibit unstable expressions under different experiment conditions, which necessitates the use of multiple reference genes [36, 38, 41, 48, 49]. As demonstrated in our work, two stably expressed reference genes can be employed in experiments, and more than two stably expressed reference genes may be necessary when there are more variations under some conditions. Our study showed that a combination of multiple reference genes could improve the accuracy and reliability of normalization to some extents. One issue arising from the use of multiple reference genes is the impacts of the potential interaction among reference genes [43]. Positive or negative interactions among reference gene expressions, such as functional gene associations or linkage disequilibrium among expressed alleles, might alter their use for normalizing target gene expressions. However, when the reference genes are linearly additive in their expressions, use of multiple reference genes could enhance the power for normalization under various experiment conditions. Therefore, how to select multiple reference genes remains to be clarified with deliberate experiment designs.

## Conclusion

Our work represents the first report in selecting and validating the expression stability of a set of candidate reference genes in *M*. *oleifera*. The results also highlight the significance of selecting proper reference genes for chosen experiments in this species. Furthermore, more than one reference gene is suggested for more accurate normalization in RT-qPCR analysis. The expression normalization results from three isotypes of the target *SOD* gene further confirmed the necessity of reliable reference genes. All these findings could aid in accurately quantifying the expression profiles of multiple target genes under different conditions in *M*. *oleifera*.

## Supporting Information

S1 Fig Amplifications of three *SOD* genes each with a different size estimated from markers. (DOCX)

S2 Fig Dissociation curves for eighteen candidate reference genes in *Moringa oleifera*. (DOCX)

S1 Table Superoxide dismutase genes examined in *Moringa oleifera*. (DOCX)

S2 Table Orders of the top-ten candidate reference genes from the analyses with geNorm, NormFinder and BestKeeper algorithms. G, N, B and O represent the orders from the analyses with geNorm, NormFinder, BestKeeper and Overall ranking, respectively.
(DOCX)

## References

[1] Anwar F, Latif S, Ashraf M, Gilani AH. *Moringa oleifera*: A food plant with multiple medicinal uses. Phytotherapy Research. 2007; 21(1): 17–25. 10.1002/ptr.2023 . 17089328

[2] Gismondi A, Canuti L, Impei S, Di Marco G, Kenzo M, Colizzi V, et al Antioxidant extracts of African medicinal plants induce cell cycle arrest and differentiation in B16F10 melanoma cells. International Journal of Oncology. 2013; 43(3): 956–64. 10.3892/ijo.2013.2001 . 23817892

[3] Verma AR, Vijayakumar M, Mathela CS, Rao CV. In vitro and in vivo antioxidant properties of different fractions of *Moringa oleifera* leaves. Food and Chemical Toxicology. 2009; 47(9): 2196–201. 10.1016/j.fct.2009.06.005 . 19520138

[4] Foidl N, Makkar H P S and Becker K. The potential of Moringa oleifera for agricultural and industrial uses. What development potential for Moringa products? October 20th—November 2nd 2001; In: Dar Es Salaam.

[5] Smit R, Du Toit ES, Vorster BJ. RAPD and SSR genetic diversity analysis of *Moringa oleifera*. South African Journal of Botany. 2013; 86: 182–. 10.1016/j.sajb.2013.02.162 .

[6] Tian Y, Zeng Y, Zhang J, Yang CG, Yan L, Wang XJ, et al High quality reference genome of drumstick tree (*Moringa oleifera* Lam.), a potential perennial crop. Sci China Life Sci. 2015;58(7):627–38. 10.1007/s11427-015-4872-x 26032590

[7] Bustin SA, Benes V, Garson JA, Hellemans J, Huggett J, Kubista M, et al The MIQE guidelines: minimum information for publication of quantitative Real-Time PCR experiments. Clin Chem. 2009; 55(4): 611–22. 10.1373/clinchem.2008.112797 19246619

[8] Bustin SA. Quantification of mRNA using real-time reverse transcription PCR (RT-PCR): Trends and Problems. J Mol Endocrinol. 2002; 29(1). . 10.1677/jme.0.0290023 12200227

[9] Artico S, Nardeli SM, Neto OBO, Grossi-de-Sa MF, Alves-Ferreira M. Identification and evaluation of new reference genes in *Gossypium hirsutum* for accurate normalization of real-time quantitative RT-PCR data. BMC Plant Biol. 2010; 10 . 10.1186/1471-2229-10-49 PMC2923523 20302670

[10] Die JV, Roman B, Nadal S, Gonzalez-Verdejo CI. Evaluation of candidate reference genes for expression studies in *Pisum sativum* under different experimental conditions. Planta. 2010; 232(1): 145–53. 10.1007/s00425-010-1158-1 20379832

[11] Schmid H, Cohen CD, Henger A, Irrgang S, Schlondorff D, Kretzler M. Validation of endogenous controls for gene expression analysis in microdissected human renal biopsies. Kidney Int. 2003; 64(1): 356–60. . 12787429 10.1046/j.1523-1755.2003.00074.x

[12] Vandesompele J, De Preter K, Pattyn F, Poppe B, Van Roy N, De Paepe A, et al Accurate normalization of real-time quantitative RT-PCR data by geometric averaging of multiple internal control genes. Genome Biology. 2002; 3(7): Research0034-Research. Medline:. 12184808 10.1186/gb-2002-3-7-research0034 PMC126239

[13] Volkov RA, Panchuk II, Schoffl F. Heat-stress-dependency and developmental modulation of gene expression: the potential of house-keeping genes as internal standards in mRNA expression profiling using real-time RT-PCR. J Exp Bot. 2003; 54(391): 2343–9. . 14504302 10.1093/jxb/erg244

[14] Wan HJ, Zhao ZG, Qian CT, Sui YH, Malik AA, Chen JF. Selection of appropriate reference genes for gene expression studies by quantitative real-time polymerase chain reaction in cucumber. Anal Biochem. 2010; 399(2): 257–61. 10.1016/j.ab.2009.12.008 20005862

[15] Czechowski T, Stitt M, Altmann T, Udvardi MK, Scheible WR. Genome-wide identification and testing of superior reference genes for transcript normalization in arabidopsis. Plant Physiol. 2005; 139(1):5–17. . 16166256 10.1104/pp.105.063743 PMC1203353

[16] Libault M, Thibivilliers S, Bilgin DD, Radwan O, Benitez M, Clough SJ, et al Identification of four soybean reference genes for gene expression normalization. Plant Genome-Us. 2008; 1(1): 44–54. .

[17] Exposito-Rodriguez M, Borges AA, Borges-Perez A, Perez JA. Selection of internal control genes for quantitative real-time RT-PCR studies during tomato development process. BMC Plant Biol. 2008; 8 . 10.1186/1471-2229-8-131 PMC2629474 19102748

[18] Kim BR, Nam HY, Kim SU, Kim SI, Chang YJ. Normalization of reverse transcription quantitative-PCR with housekeeping genes in rice. Biotechnol Lett. 2003; 25(21): 1869–72. . 14677714 10.1023/a:1026298032009

[19] Schmidt GW, Delaney SK. Stable internal reference genes for normalization of real-time RT-PCR in tobacco (*Nicotiana tabacum*) during development and abiotic stress. Mol Genet Genomics. 2010; 283(3): 233–41. 10.1007/s00438-010-0511-1 20098998

[20] Radonic A, Thulke S, Mackay IM, Landt O, Siegert W, Nitsche A. Guideline to reference gene selection for quantitative real-time PCR. Biochem Bioph Res Co. 2004; 313(4): 856–62. . 10.1016/j.bbrc.2003.11.177 14706621

[21] Chen L, Zhong H-y, Kuang J-f, Li J-g, Lu W-j, Chen J-y. Validation of reference genes for RT-qPCR studies of gene expression in banana fruit under different experimental conditions. Planta. 2011; 234(2):377–90. 10.1007/s00425-011-1410-3 . 21505864

[22] Huggett J, Dheda K, Bustin S, Zumla A. Real-time RT-PCR normalisation; strategies and considerations. Genes Immun. 2005; 6(4): 279–84. . 15815687 10.1038/sj.gene.6364190

[23] Lovdal T, Lillo C. Reference gene selection for quantitative real-time PCR normalization in tomato subjected to nitrogen, cold, and light stress. Anal Biochem. 2009; 387(2): 238–42. 10.1016/j.ab.2009.01.024 19454243

[24] Zhu XY, Li XP, Chen WX, Chen JY, Lu WJ, Chen L, et al Evaluation of new reference genes in papaya for accurate transcript normalization under different experimental conditions. Plos One. 2012; 7(8). . 10.1371/journal.pone.0044405 PMC3432124 22952972

[25] Nicot N, Hausman JF, Hoffmann L, Evers D. Housekeeping gene selection for real-time RT-PCR normalization in potato during biotic and abiotic stress. J Exp Bot. 2005; 56(421): 2907–14. . 16188960 10.1093/jxb/eri285

[26] Gutierrez L, Mauriat M, Guenin S, Pelloux J, Lefebvre JF, Louvet R, et al The lack of a systematic validation of reference genes: a serious pitfall undervalued in reverse transcription-polymerase chain reaction (RT-PCR) analysis in plants. Plant Biotechnol J. 2008; 6(6): 609–18. 10.1111/j.1467-7652.2008.00346.x 18433420

[27] Saini RK, Prashanth KVH, Shetty NP, Giridhar P. Elicitors, SA and MJ enhance carotenoids and tocopherol biosynthesis and expression of antioxidant related genes in *Moringa oleifera* Lam. leaves. Acta Physiol Plant. 2014; 36(10): 2695–704. .

[28] Mittler R. Oxidative stress, antioxidants and stress tolerance. Trends Plant Sci. 2002; 7(9): 405–10. . 12234732 10.1016/s1360-1385(02)02312-9

[29] Mohamed E, Matsuda R, El-khatib AA, Takechi K, Takano H, Takio S. Characterization of the superoxide dismutase genes of the halophyte *Suaeda maritima* in Japan and Egypt. Plant Cell Rep. 2015; 34(12): 2099–110. 10.1007/s00299-015-1854-1 26267391

[30] Alscher RG, Erturk N, Heath LS. Role of superoxide dismutases (SODs) in controlling oxidative stress in plants. J Exp Bot. 2002; 53(372): 1331–41. . 11997379

[31] Andersen CL, Jensen JL, Orntoft TF. Normalization of real-time quantitative reverse transcription-PCR data: A model-based variance estimation approach to identify genes suited for normalization, applied to bladder and colon cancer data sets. Cancer Res. 2004; 64(15):5245–50. . 15289330 10.1158/0008-5472.CAN-04-0496

[32] Pfaffl MW, Tichopad A, Prgomet C, Neuvians TP. Determination of stable housekeeping genes, differentially regulated target genes and sample integrity: BestKeeper-Excel-based tool using pair-wise correlations. Biotechnol Lett. 2004; 26(6): 509–15. . 15127793 10.1023/b:bile.0000019559.84305.47

[33] Gomez JM, Jimenez A, Olmos E, Sevilla F. Location and effects of long-term NaCl stress on superoxide dismutase and ascorbate peroxidase isoenzymes of pea (*Pisum sativum* cv. Puget) chloroplasts. J Exp Bot. 2004; 55(394): 119–30. . 14676290 10.1093/jxb/erh013

[34] Gill SS, Anjum NA, Gill R, Yadav S, Hasanuzzaman M, Fujita M, et al Superoxide dismutase-mentor of abiotic stress tolerance in crop plants. Environ Sci Pollut R. 2015;22(14):10375–94. . 10.1007/s11356-015-4532-5 25921757

[35] Xu M, Zhang B, Su XH, Zhang SG, Huang MR. Reference gene selection for quantitative Real-Time polymerase chain reaction in populus. Anal Biochem. 2011;408(2):337–9. 10.1016/j.ab.2010.08.044 20816740

[36] Dheda K, Huggett JF, Bustin SA, Johnson MA, Rook G, Zumla A. Validation of housekeeping genes for normalizing RNA expression in real-time PCR. Biotechniques. 2004; 37(1): 112–+. . 15283208 10.2144/04371RR03

[37] Brunner AM, Yakovlev IA, Strauss SH. Validating internal controls for quantitative plant gene expression studies. BMC Plant Biol. 2004;4(1):1. 15317655 10.1186/1471-2229-4-14 PMC515301

[38] Reid KE, Olsson N, Schlosser J, Peng F, Lund ST. An optimized grapevine RNA isolation procedure and statistical determination of reference genes for real-time RT-PCR during berry development. BMC Plant Biol. 2006; 6 10.1186/1471-2229-6-27 PMC1654153 17105665

[39] Lee PD, Sladek R, Greenwood CMT, Hudson TJ. Control genes and variability: Absence of ubiquitous reference transcripts in diverse mammalian expression studies. Genome Research. 2002; 12(2): 292–7. . 11827948 10.1101/gr.217802 PMC155273

[40] Suzuki T, Higgins P, Crawford D. Control selection for RNA quantitation. Biotechniques. 2000; 29(2):332–7. 10948434 10.2144/00292rv02

[41] Tricarico C, Pinzani P, Bianchi S, Paglierani M, Distante V, Pazzagli M, et al Quantitative real-time reverse transcription polymerase chain reaction: normalization to rRNA or single housekeeping genes is inappropriate for human tissue biopsies. Anal Biochem. 2002; 309(2): 293–300. . 12413463 10.1016/s0003-2697(02)00311-1

[42] Buckland PR. The importance and identification of regulatory polymorphisms and their mechanisms of action. Bba-Mol Basis Dis. 2006; 1762(1):17–28. . 10.1016/j.bbadis.2005.10.004 16297602

[43] Silver N, Best S, Jiang J, Thein SL. Selection of housekeeping genes for gene expression studies in human reticulocytes using real-time PCR. BMC Mol Biol. 2006; 7 10.1186/1471-2199-7-33 PMC1609175 17026756

[44] Ando H, Nakano K, Ushijima K, Kurokawa S, Washino S, Hosohata K, et al Influence of genetic polymorphisms of multidrug and toxin extrusion protein 1 on its mRNA expression in peripheral blood cells. J Pharmacol Sci. 2016 10.1016/j.jphs.2016.03.002 27025966

[45] Kimura M, Ohta T. On some principles governing molecular evolution. Proceedings of the National Academy of Sciences. 1974; 71(7): 2848–52. 10.1073/pnas.71.7.2848 PMC388569 4527913

[46] Zhang L, He LL, Fu QT, Xu ZF. Selection of reliable reference genes for gene expression studies in the biofuel plant *Jatropha curcas* using Real-Time quantitative PCR. Int J Mol Sci. 2013; 14(12): 24338–54. 10.3390/ijms141224338 24351820 PMC3876114

[47] Kumar V, Sharma R, Trivedi PC, Vyas GK, Khandelwal V. Traditional and novel references towards systematic normalization of qRT-PCR data in plants. Aust J Crop Sci. 2011; 5(11): 1455–68. .

[48] Neuvians TAP, Gashaw I, Sauer CG, von Ostau C, Kliesch S, Bergmann M, et al Standardization strategy for quantitative PCR in human seminoma and normal testis. J Biotechnol. 2005; 117(2): 163–71. . 15823405 10.1016/j.jbiotec.2005.01.011

[49] Shivhare R, Lata C. Selection of suitable reference genes for assessing gene expression in pearl millet under different abiotic stresses and their combinations. Sci Rep-Uk. 2016; 6 . 10.1038/srep23036 PMC4789795 26972345

