# Supplementary figures and images for: Screening Reliable Reference Genes for RT-qPCR Analysis of Gene Expression in Moringa oleifera
Source: PLoS One. 2016 Aug 19;11(8):e0159458. doi: 10.1371/journal.pone.0159458 (PMC4991797; doi:10.1371/journal.pone.0159458)

**S1 Fig. Amplifications of three *SOD* genes each with a different size estimated from markers**


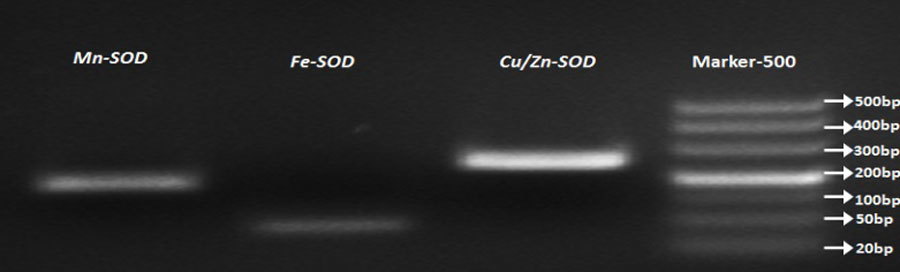

Supplement: S1 Fig — (DOCX) [file pone.0159458.s001.docx]

**S2 Fig. Dissociation curves for eighteen candidate reference genes in *Moringa oleifera***


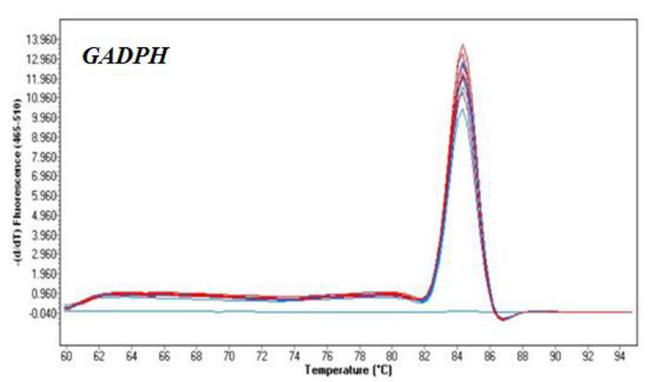
**
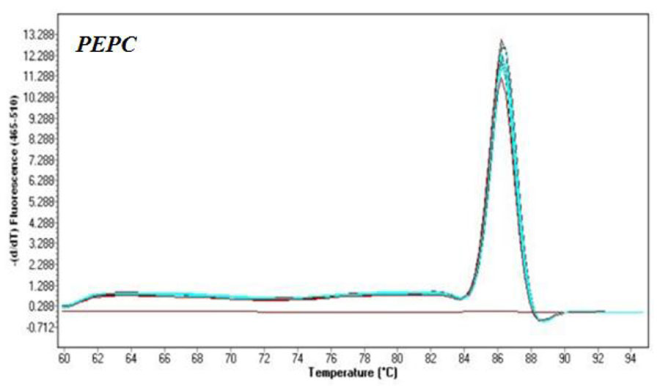

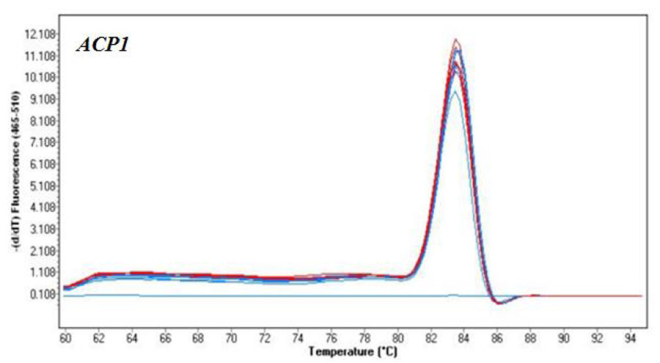

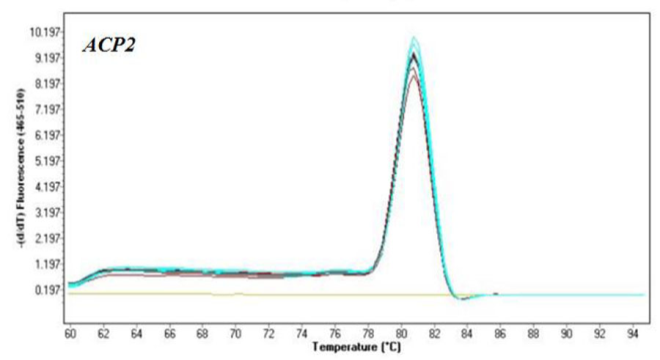

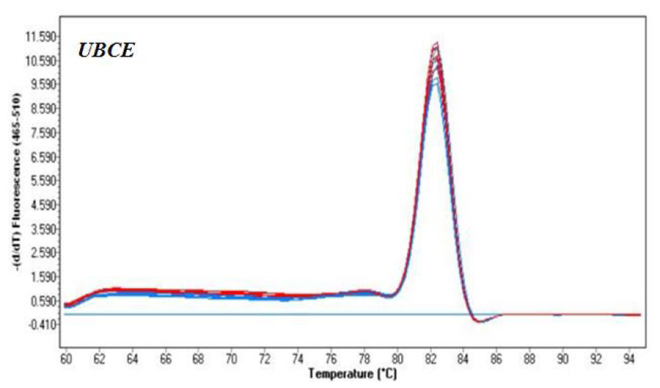

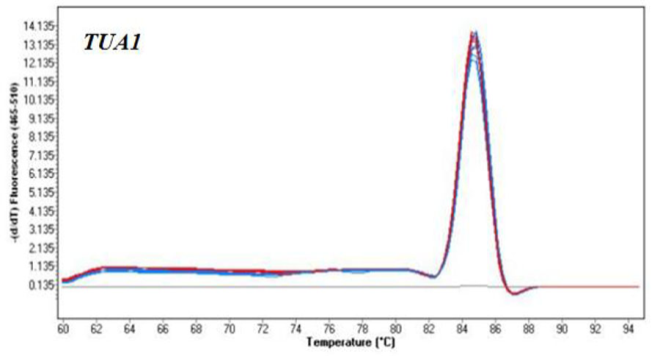

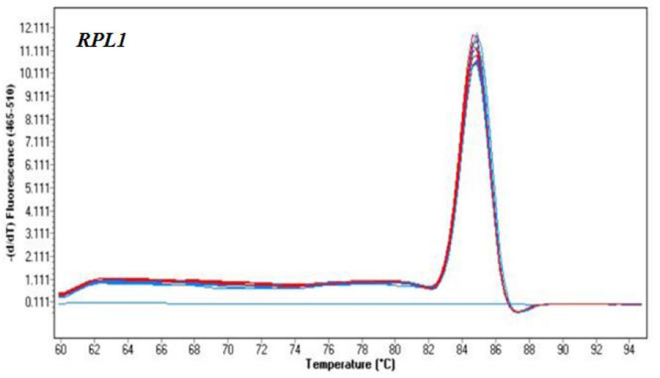

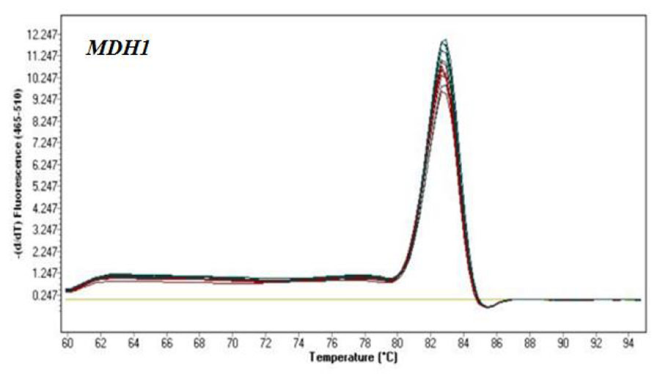

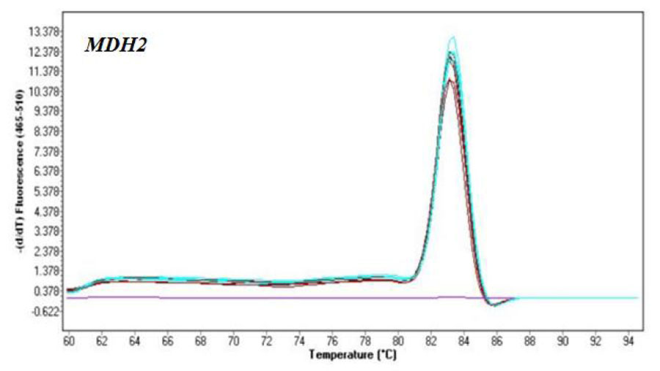

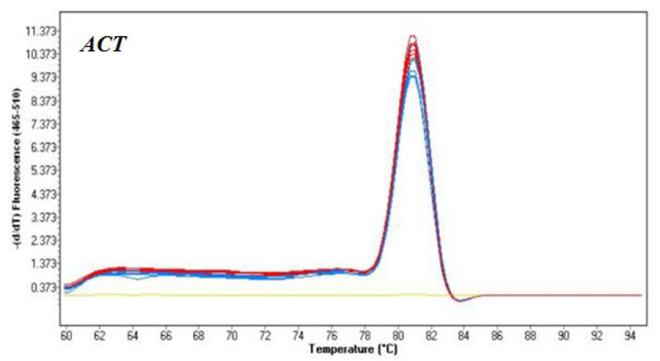

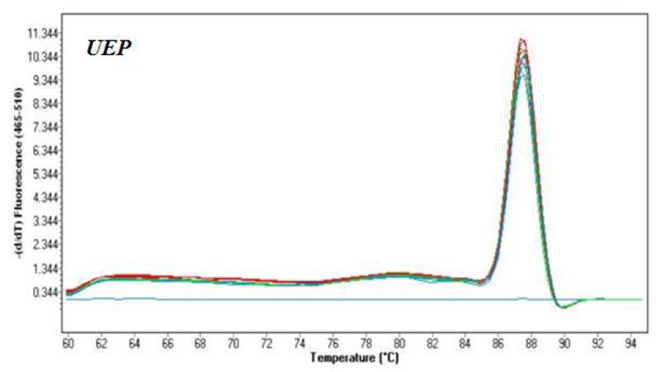

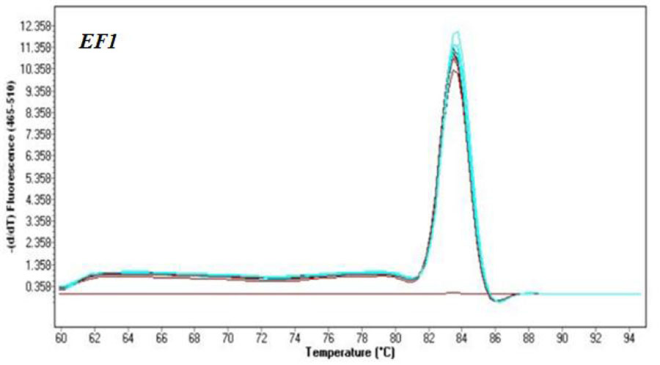

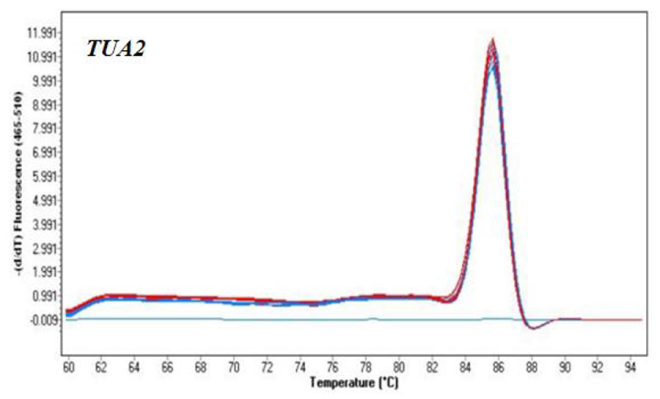

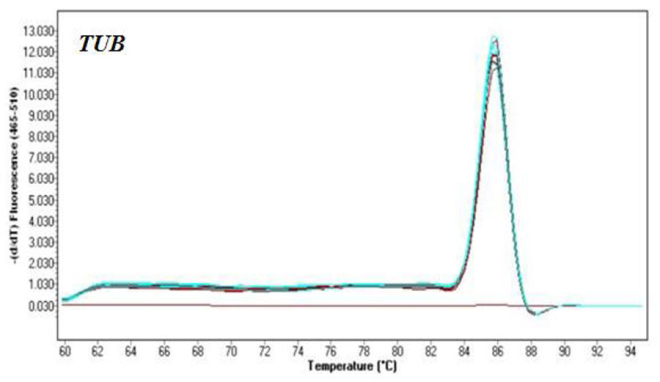

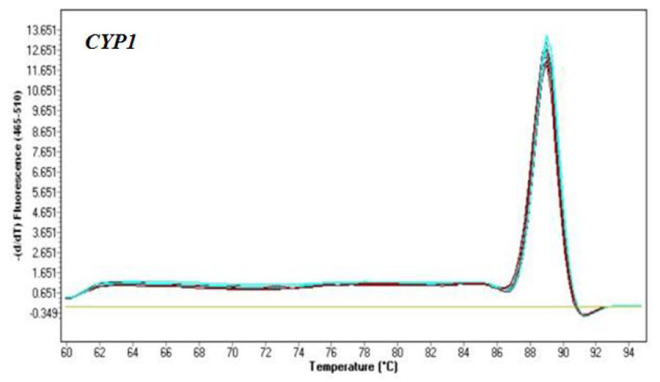

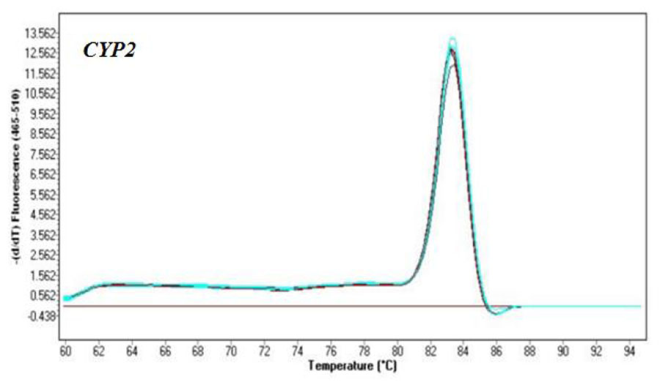

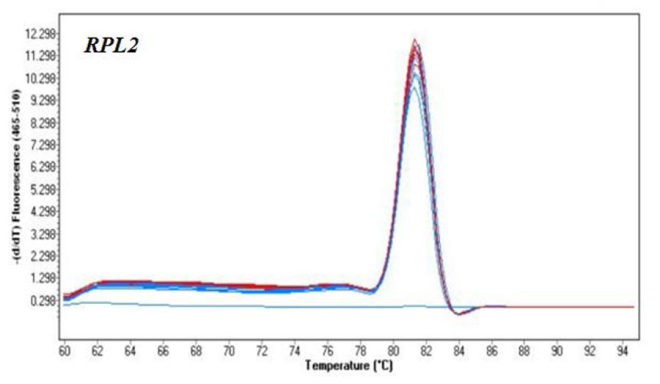

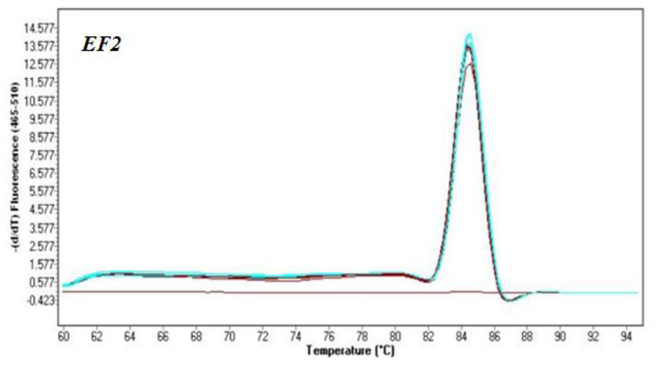
**

Supplement: S2 Fig — (DOCX) [file pone.0159458.s002.docx]
