# Supplementary material for: Screening Reliable Reference Genes for RT-qPCR Analysis of Gene Expression in Moringa oleifera
Source: PLoS One. 2016 Aug 19;11(8):e0159458. doi: 10.1371/journal.pone.0159458 (PMC4991797; doi:10.1371/journal.pone.0159458)
Supplement: S1 Table — (DOCX) [file pone.0159458.s003.docx]

**S1 Table. Superoxide dismutase genes examined in *Moringa oleifera***

| **Genes** | **Abbreviation** | **Primer sequences (Forward/reverse)** | **Amplicon length(bp)** |
| --- | --- | --- | --- |
| Manganese superoxide dismutase | Mn-SOD | AGAGCAGCACCTTCAGCAT/  CTACTCCAAAGCCCCATCAAA | 188 |
| Iron superoxide dismutase | Fe-SOD | TTCTATGATGCTTTCCCCAATG/  AGCCACCTCCATATCCGT | 84 |
| Copper and zinc superoxide dismutase | Cu/Zn-SOD | GAGGTAGCAAAGAAGGGGTAA/  GGATTGAAATGAGGTCCAGTG | 244 |
