# Supplementary material for: Screening Reliable Reference Genes for RT-qPCR Analysis of Gene Expression in Moringa oleifera
Source: PLoS One. 2016 Aug 19;11(8):e0159458. doi: 10.1371/journal.pone.0159458 (PMC4991797; doi:10.1371/journal.pone.0159458)
Supplement: S2 Table — G, N, B and O represent the orders from the analyses with geNorm, NormFinder, BestKeeper and Overall ranking, respectively. (DOCX) [file pone.0159458.s004.docx]

**S2 Table. Orders of the top-ten candidate reference genes from the analyses with geNorm, NormFinder and BestKeeper algorithms. G, N, B and O represent the orders from the analyses with geNorm, NormFinder, BestKeeper and Overall ranking, respectively.**

| **Developmental stages** | | | | |  | **Various tissues** | | | | |  | **Chilling** | | | | |
| --- | --- | --- | --- | --- | --- | --- | --- | --- | --- | --- | --- | --- | --- | --- | --- | --- |
| Genes | G | N | B | O |  | Genes | G | N | B | O |  | Genes | G | N | B | O |
| *ACT* | 1 | 1 | 1 | 1 |  | *TUB* | 1 | 8 | 3 | 2 |  | *TUA2* | 1 | 2 | 4 | 1 |
| *RPL1* | 2 | 3 | 6 | 2 |  | *ACT* | 2 | 6 | 6 | 3 |  | *TUB* | 2 | 1 | 5 | 2 |
| *EF1* | 3 | 6 | 7 | 5 |  | *TUA1* | 3 | 9 | 2 | 4 |  | *MDH2* | 3 | 5 | 3 | 3 |
| *EF2* | 4 | 5 | 8 | 6 |  | *ACP2* | 4 | 5 | 1 | 1 |  | *CYP2* | 4 | 8 | 10 | 7 |
| *MDH2* | 5 | 2 | 4 | 3 |  | *EF1* | 5 | 2 | 7 | 5 |  | *MDH1* | 5 | 3 | 9 | 6 |
| *CYP2* | 6 | 4 | 3 | 4 |  | *CYP1* | 6 | 1 | 8 | 6 |  | *PEPC* | 6 | 7 | 1 | 4 |
| *UEP* | 7 | 9 | 2 | 7 |  | *ACP1* | 7 | 3 | 10 | 7 |  | *RPL1* | 7 | 10 | 7 | 9 |
| *ACP2* | 8 | 8 | 5 | 8 |  | *UEP* | 8 | 4 | 9 | 8 |  | *ACP1* | 8 | 4 | 2 | 5 |
| *ACP1* | 9 | 7 | 10 | 9 |  | *RPL1* | 9 | 7 | 5 | 9 |  | *ACT* | 9 | 6 | 8 | 8 |
| *RPL2* | 10 | 10 | 9 | 10 |  | *TUA2* | 10 | 10 | 4 | 10 |  | *ACP2* | 10 | 9 | 6 | 10 |
| **High temperature** | | | | |  | **NaCl** | | | | |  | **PEG** | | | | |
| *ACP1* | 1 | 2 | 8 | 2 |  | *ACP1* | 1 | 6 | 3 | 3 |  | *EF2* | 1 | 1 | 2 | 1 |
| *RPL2* | 2 | 1 | 6 | 1 |  | *CYP1* | 2 | 5 | 5 | 4 |  | *RPL2* | 2 | 2 | 7 | 2 |
| *RPL1* | 3 | 5 | 7 | 6 |  | *MDH2* | 3 | 2 | 1 | 1 |  | *ACP1* | 3 | 4 | 5 | 4 |
| *MDH1* | 4 | 3 | 4 | 3 |  | *ACP2* | 4 | 1 | 2 | 2 |  | *MDH1* | 4 | 3 | 4 | 3 |
| *TUA1* | 5 | 7 | 2 | 4 |  | *EF2* | 5 | 3 | 7 | 5 |  | *ACP2* | 5 | 6 | 1 | 5 |
| *UEP* | 6 | 4 | 5 | 7 |  | *RPL1* | 6 | 4 | 8 | 6 |  | *MDH2* | 6 | 5 | 3 | 6 |
| *TUB* | 7 | 6 | 1 | 5 |  | *MDH1* | 7 | 7 | 4 | 7 |  | *UEP* | 7 | 8 | 6 | 7 |
| *CYP1* | 8 | 9 | 9 | 9 |  | *ACT* | 8 | 8 | 9 | 9 |  | *RPL1* | 8 | 7 | 8 | 8 |
| *ACP2* | 9 | 10 | 10 | 10 |  | *RPL2* | 9 | 9 | 6 | 8 |  | *GADPH* | 9 | 10 | 9 | 9 |
| *EF2* | 10 | 8 | 3 | 8 |  | *UEP* | 10 | 10 | 10 | 10 |  | *ACT* | 10 | 9 | 10 | 10 |
| **NaCl and PEG** | | | | |  | **Chilling and high temperature** | | | | |  | **All samples** | | | | |
| *MDH2* | 1 | 4 | 4 | 2 |  | *ACP1* | 1 | 1 | 6 | 1 |  | *ACP2* | 1 | 5 | 2 | 2 |
| *ACP2* | 2 | 3 | 1 | 1 |  | *RPL2* | 2 | 4 | 7 | 4 |  | *RPL1* | 2 | 8 | 3 | 4 |
| *EF2* | 3 | 1 | 5 | 3 |  | *RPL1* | 3 | 3 | 5 | 2 |  | *UEP* | 3 | 2 | 1 | 1 |
| *ACP1* | 4 | 2 | 7 | 5 |  | *ACP2* | 4 | 9 | 8 | 7 |  | *ACT* | 4 | 3 | 4 | 3 |
| *RPL1* | 5 | 5 | 2 | 4 |  | *MDH1* | 5 | 2 | 4 | 3 |  | *ACP1* | 5 | 6 | 5 | 6 |
| *RPL2* | 6 | 6 | 6 | 7 |  | *ACT* | 6 | 7 | 10 | 9 |  | *MDH2* | 6 | 1 | 6 | 5 |
| *UEP* | 7 | 7 | 3 | 6 |  | *UEP* | 7 | 6 | 2 | 6 |  | *EF1* | 7 | 9 | 7 | 7 |
| *ACT* | 8 | 8 | 9 | 8 |  | *TUA1* | 8 | 5 | 1 | 5 |  | *GADPH* | 8 | 10 | 8 | 9 |
| *GADPH* | 9 | 9 | 8 | 9 |  | *CYP1* | 9 | 10 | 9 | 10 |  | *RPL2* | 9 | 7 | 10 | 10 |
| *EF1* | 10 | 10 | 10 | 10 |  | *EF2* | 10 | 8 | 3 | 8 |  | *CYP1* | 10 | 4 | 9 | 8 |
